# Understanding the Role of Social Media–Based Mental Health Support Among College Students: Survey and Semistructured Interviews

**DOI:** 10.2196/24512

**Published:** 2021-07-12

**Authors:** Piper Vornholt, Munmun De Choudhury

**Affiliations:** 1 School of Interactive Computing College of Computing Georgia Institute of Technology Atlanta, GA United States

**Keywords:** college mental health, social media, social support, mobile phone

## Abstract

**Background:**

Mental illness is a growing concern within many college campuses. Limited access to therapy resources, along with the fear of stigma, often prevents students from seeking help. Introducing supportive interventions, coping strategies, and mitigation programs might decrease the negative effects of mental illness among college students.

**Objective:**

Many college students find social support for a variety of needs through social media platforms. With the pervasive adoption of social media sites in college populations, in this study, we examine whether and how these platforms may help meet college students’ mental health needs.

**Methods:**

We first conducted a survey among 101 students, followed by semistructured interviews (n=11), of a large public university in the southeast region of the United States to understand whether, to what extent, and how students appropriate social media platforms to suit their struggle with mental health concerns. The interviews were intended to provide comprehensive information on students’ attitudes and their perceived benefits and limitations of social media as platforms for mental health support.

**Results:**

Our survey revealed that a large number of participating students (71/101, 70.3%) had recently experienced some form of stress, anxiety, or other mental health challenges related to college life. Half of them (52/101, 51.5%) also reported having appropriated some social media platforms for self-disclosure or help, indicating the pervasiveness of this practice. Through our interviews, we obtained deeper insights into these initial observations. We identified specific academic, personal, and social life stressors; motivations behind social media use for mental health needs; and specific platform affordances that helped or hindered this use.

**Conclusions:**

Students recognized the benefits of social media in helping connect with peers on campus and promoting informal and candid disclosures. However, they argued against complete anonymity in platforms for mental health help and advocated the need for privacy and boundary regulation mechanisms in social media platforms supporting this use. Our findings bear implications for informing campus counseling efforts and in designing social media–based mental health support tools for college students.

## Introduction

### Background

Students’ mental health problems are pervasive and serious. In a survey conducted a few years ago, 32.9% of college students answered that they were diagnosed with or treated by a professional for a number of mental health–related issues, such as anorexia, depression, and panic attacks. [1,2]. In the same survey, 60% reported feeling overwhelming anxiety in the last 12 months, and 57% of students answered that the overall level of stress they experienced was higher than the stress experienced by their nonstudent peers. Such mental health concerns can negatively impact students’ academic success and their career development [3].

However, many college students with mental health conditions are not seeking help because of stigma they would face from family, friends, faculty, or other students [4]. The National Alliance on Mental Health surveyed college students and found that 50% of students who left school because of mental health reasons did not access mental health services and support offered on campus [5]. The National Survey of Counseling Center Directors similarly revealed that 87% of students who died by suicide in 2010 never sought assistance from counseling or mental health services provided at their campuses [6].

Therefore, it has been posited that the introduction of supportive interventions, coping strategies, and mitigation programs might decrease the negative effects of mental illness in college students, especially among those who might be hesitant to utilize formal psychological services on campus [7-9]. These approaches can also counteract and compensate for limited access to psychiatric facilities and systems for treating and managing mental health conditions in college students, such as those centered around education and therapy [10]. In particular, social support is recognized as a key ingredient in managing mental health [11] and preventing anxiety and depression from becoming a serious concern [12]. Support is particularly critical to overcoming the burden of stigma among college students who find themselves in a new and unfamiliar environment. They may fear that self-disclosing their mental health challenges with counseling services can lead to biased or negative judgments about them or compromise their privacy [13]. Avoidance factors, such as fear of treatment, desire to conceal distress or personal information, and the desire to avoid experiencing increased painful feelings during therapy, may additionally impact college students’ decisions to not make use of formal psychological services [14]. Talking to a peer coach or student counselor can help students find social support through alternative and informal means; research has found that peer support specialists can go beyond treatment as usual and use different training and skills to support recovery in conjunction with professionals like therapists, social workers, and psychiatrists [15]. However, not every student feels comfortable seeking support in person within their campus communities. Moreover, college students move across towns, states, and even countries to come to colleges where they often know no one. Although they may still have some support from family and friends back home, finding new in-person support at school can be challenging and time-consuming for students dealing with mental illness [16].

The web is increasingly used to seek and share health information on the web [17]. In particular, social media platforms have begun to offer new opportunities to meet the mental health needs of college students and serve as a means of support [18]. In an early work, Eysenbach et al [19] reported that web-based communities could be seen as platforms to deliver mental health and social support interventions—they often have the function and character of self-support offline groups. A key aspect of these communities is providing members with access to other people with similar challenging conditions [20]. Adopting Cutrona and Suhr schema called *Social Support Behavioral Code* for understanding and assessing support along the dimensions of emotional support and informational support [21], prior research has found that members of web-based communities receive emotional support either directly, through empathetic messages, or indirectly, by being exposed to others having similar experiences [22]. They also gain informational support by receiving helpful information and advice related to treatment and medication, identifying possible explanations for their problems, and building social capital [23].

Currently, more than 90% of young adults or individuals of college-going age use social media [24]. A study of a student-centered social media site, SpartanConnect (a website specific to Michigan State University), showed that the website increased students’ perception of a diverse social support system [25]. With a variety of social media platforms available to them, students have many options for finding social support as they transition into college. However, are students making use of these social media platforms to address their mental health needs? What benefits and challenges do they experience in using these platforms to find mental health support?

### Objectives

This paper presents a formative study to *explore and understand the role of social media technologies as a complementary source of social support to college students experiencing mental health concerns*. Adopting a two-prong approach, we accomplish 2 goals: (1) we first surveyed a large public university located in the southeast of the United States to identify the extent to which students are appropriating social media platforms to suit their mental health needs. (2) Next, we conducted semistructured interviews with students who identified themselves as struggling with mental health concerns. The interviews sought to describe and provide insights into college students’ attitudes and their perceived benefits and limitations of social media as platforms for mental health support.

## Methods

### Survey

We began by conducting a web-based survey aimed at students currently enrolled full-time at a large public university in the southeast of the United States. Our goal was to assess how attributes of mental well-being are related to students’ social media use and to what extent they were appropriating these platforms to cater to their mental health needs. The survey was approved by the authors’ institutional review board.

To gauge student well-being, we used 4 well-validated measures, selection for which was guided by prior research on college student mental health [12]. These measures assessed both objective manifestations of different mental health challenges (eg, anxiety and stress) common in the college student demographic [16] (stress and anxiety are among the most common mental health concerns among college students [9]) as well as identify factors that affect (eg, college environment) or are affected by mental health challenges (eg, self-esteem). A variety of prior research has explored how social-ecological factors affect students’ mental health [8,25]. Accordingly, we included the following: (1) the College Adjustment Test (CAT) [26] (Cronbach *α*=.79), (2) Generalized Anxiety Disorder-7 scale [27] (Cronbach *α*=.83), (3) Perceived Stress Scale [28] (Cronbach *α*=.87), and (4) the Rosenberg Self-Esteem Scale [29] (Cronbach *α*=.86). To evaluate levels of social support, we included the medical outcomes study (MOS) social support survey [30] (Cronbach *α*=.97). We also included a final measure, borrowing questions from the Facebook Intensity Scale [31] (Cronbach *α*=.83) and the Zammit Social Media Questionnaire [32] (Cronbach *α*=.96), which gauged participants’ social media usage and behavior—this was because of our focus on examining the role played by social technologies in providing support around mental illnesses. Along with these measures, we included a single question on the extent to which participants used social media to seek help, advice, and support for their mental health; this included a 5-point Likert scale where 1-2 indicated little or no use, 3 indicated moderate use, and 4-5 indicated high use. Finally, our survey contained open-ended questions that aimed to identify how students may or may not use social media to gain social support around mental health needs, such as questions on their intent and motivation, what methods they use to manage their mental health (eg, stress or anxiety) on social media, how they appropriate social media to cope with stress or anxiety, and any perceived barriers to mental health support seeking on social media. The survey also collected basic academic and demographic information, including academic year, major, sex, and ethnicity.

Our selection criteria included any undergraduate or graduate students aged 18 to 24 years enrolled full-time at the university at the time of the survey; part-time students were excluded as they likely experienced a significantly different set of mental health stressors. That said, even among those included, although the 2 student groups (undergraduate and graduate) may experience slightly different sets of mental health stressors, we recruited from both populations as a way of demonstrating the feasibility and role of web-based social support in mental health as well as reaching a large and diverse population. We used both online and offline recruitment strategies. We posted the survey on the university’s Reddit community, the campus YikYak (a now deprecated hyperlocal social media platform), Twitter (with the university hashtag), various public and private Facebook student groups, personal Twitter and Facebook profiles, in-person word-of-mouth advertisements, and flyers in different buildings around the campus, including the counseling center on the university campus. Each participant was compensated for their time using a US $10 gift card. Multimedia Appendix 1 includes our survey recruitment ad.

### Interviews

The survey provided a way to examine the patterns of social media use for individuals who choose or do not choose to seek mental health help in social media. As surveys cannot provide nuanced, subjective perceptions and opinions on *why* and *how* these platforms are being appropriated for mental health needs, we conducted the following interview study. We adopted a top-down and bottom-up approach to develop a semistructured in-person interview protocol. The top-down approach involved referring social science literature on how support is appropriated by college students to manage and overcome mental health challenges [8], particularly around identifying specific personal and ecologically grounded environmental stressors [5,6], and social media literature that explained how design (or affordances), underlying norms and conventions, identity choices, self-disclosure behaviors, and community interactions shape people’s help and information-seeking attitudes on the web [33]. With these theorizations and conceptualizations, we framed interview questions focusing on the web-based aspect of social support. In the bottom-up approach, we revisited the open-ended responses in our survey to identify issues and topics that could use more elaborate discussion. On merging the outcomes of the two approaches, the final protocol focused on the following aspects: (1) stressors or sources of anxiety and mental health concerns students face; (2) the role that social media plays in satisfying students’ mental health needs; and (3) the affordances of social media sites that they identify to be most critical to their success as a platform for mental health disclosure and support. Our interview guide is included in Multimedia Appendix 2.

Our selection criteria included full-time undergraduate students who used social media sites for mental health needs. We exclusively focused on undergraduates as the target group as our survey identified them to be most challenged with mental health concerns. Recruitment for interviews occurred in a manner similar to the survey. In addition, we met with 2 licensed psychologists at the counseling center: the assessment services coordinator and the outreach and professional development coordinator as well as the mental health student coalition group toward our recruitment efforts.

Our research team conducted semistructured interviews with 11 undergraduate students. This N was determined based on the number of interviews at which some level of theoretical saturation for the interview questions was achieved; that is, we found interviewees after the first 10 generally reiterated themes and patterns observed in the already collected data, and those interviews did not lead to drastic revisions of the themes or categories in the analysis. This practice is common in qualitative research [34]. Interviews lasted 23 to 71 minutes (median 40 minutes). Participants were told that they could stop the interview at any time and provided with a counseling information resource handout before the interview began, in case they experienced unexpected emotions as a consequence of the ensuing conversation. Each participant was compensated for their time with another US $10 Amazon gift card for the interviews. This study was approved by the authors’ institutional review board, as with the survey.

Following the interviews, the authors transcribed the interviews and used an inductive and iterative semiopen coding approach; 2 researchers separately read the transcripts and noted codes manually, relying on our survey findings and literature on self-disclosure and social support [35,36]. This step was followed by a mutual discussion in which more codes were incorporated, and the inconsistencies resolved. Our final list consisted of 32 codes, on which we had 100% agreement; interrater reliability before the mutual discussion was 0.69 (Cohen κ). Finally, the researchers used this codebook to code all transcripts and identify interpretive broader themes that captured commonalities and patterns across different codes, using grounded theory and inductive qualitative thematic analysis. Multimedia Appendix 3 includes the codes developed.

### Positionality

The research team includes researchers with backgrounds in psychology and computer science, particularly familiar with both the mental health and social support domains as well as social media systems. The team has extensive experience in qualitative and quantitative methods, spanning the past 15 years of experience in social media research and the past 8 years of research at the intersection of social media and mental health. Finally, the team had adopted participatory approaches to engage with domain stakeholders in this type of research, spanning mental health clinicians, advocacy groups, and public health organizations. This experience has been valuable in shaping the analytical approach of this qualitative study.

## Results

### Observations From the Survey

#### Overview

A total of 147 participants responded to our survey, which was active for 2 months. After removing incomplete responses, we were left with 101 responses that we used in our ensuing analysis. The removed data included survey responses completed extremely quickly (less than 5 min) and those that failed *trap* questions to complete (*σ*=16*.*2). Our final set of 101 participants included 56.4% (57) males and 40.6% (41) females, and 3% (3) others included in the survey to eliminate people who were not paying attention. On average, the survey took 37 minutes for those who preferred not to disclose their gender. In total, 60.4% (61/101) of participants indicated that they were White, 18.8% (19/101) were Asian, 7.9% (8/101) were African American, 7.9% (8/101) were Hispanic or Latino, 2% (2/101) were Native American, and 3% (3/101) were of other ethnicities. Our respondents were evenly distributed across different academic years. Across academic years, sophomores (25/101, 24.8%) were the largest group, followed by graduate students (Masters, PhD: 22/101, 21.8%; juniors: 21/101, 20.8%; freshmen: 14/101, 13.9%; and seniors: 14/101, 13.9%), and academic year not disclosed: 5% (5/101). Computing (30/101, 29.7%) and Engineering (21/101, 20.8%) were the 2 most common academic majors, with Sciences at 19.8% (20/101) and Liberal Arts at 14.9% (15/101). We ascribe this bias toward science, technology, engineering, and mathematics disciplines to the nature of the general student body at this university.

An overwhelmingly large number of participants (71/101, 70.3%) indicated feeling stressed and/or anxious from college life. Analyzing responses to the single survey question that assessed the extent to which students used social media for support seeking around their mental health, we assigned participants with responses 1-2 to the *do not use* cohort and those corresponding to responses 3-5 to the *use social media* cohort. In total, 51.5% (52/101) of participants indicated that they had used social media to find support from friends, peers, anonymous users, or others to cope with stress and/or anxiety (Table 1 for additional details on social media use). Comparing different online and offline recruitment strategies (university social media, public social media, word-of-mouth, and physical flyers and ads), we did not observe any statistically significant differences in these variables based on a one-way analysis of variance (*P*>.05).

In the remainder of this subsection, we focus on contrasting these participants with those who did not use social media for their mental health needs (49/101, 48.5%) and a number of dimensions, including their mental wellness (extent and characteristics) and how they use social media.

**Table 1:** Social media usage among survey respondents (N=101).

Usage factor		Respondents, n (%)
**Social networking sites and app use**		
	Facebook	69 (69)
	Snapchat	45 (44)
	Twitter	42 (42)
	Instagram	37 (32)
	YikYak	24 (24)
	Reddit	21 (21)
	LinkedIn	17 (17)
	Google+	14 (14)
	Tumblr	10 (10)
	Pinterest	6 (6)
	Other	2 (2)
**Hours per day spent using social media**		
	<1	19 (19)
	1-3	33 (33)
	4-6	26 (26)
	7-9	4 (4)
	≥10	18 (18)
**Primary source for using social media**		
	iPhone	57 (57)
	Android phone	54 (27)
	iPad	26 (26)
	Public computer	12 (12)
	Tablet (other than iPad)	11 (11)
	Other	9 (9)
**Purpose for using social media**		
	To become updated on events	54 (53)
	To communicate with family or friends	54 (53)
	To become updated on friends’ activities	47 (47)
	To meet new people	29 (29)
	To find people (old friends, classmates)	28 (28)
	For playing web-based games	11 (11)
	For using apps for smartphones	10 (10)
	To promote business or organization	4 (4)
	Other	2 (2)

#### Mental Health

From Table 2 and based on Mann-Whitney *U* tests, we observe that the cohort of participants who scored consistently higher on mental health issues, such as in the Perceived Stress Scale (51% more; *P*=.005) and Generalized Anxiety Disorder-7 scales (62% more; *P*<*.*001), also used social media more extensively to disclose and obtain support. This cohort that used social media for mental health needs also had lower access to social support as measured by the MOS scale (11% less; *P*=.02) and across all MOS factors. Among the various forms of support, we observed that emotional or informational support the average social media mental health help seekers expressed lower self-esteem (38% less; *P*=.009). In addition, overall college adjustment, as measured by the CAT scale, was lower in this cohort (5% less; *P*=.03). Thus, we conjecture that this cohort may not receive as much empathy, advice, or help from their existing support systems, and therefore might be appropriating web-based resources. Next, this cohort also felt higher homesickness (31% more; *P=*.006); this measure reflects the extent to which a student misses their home or friends or feels lonely at college [26]. They also expressed higher negative affect (27% more; *P*=.009) and lower positive affect (28% less; *P*=.009), as given by the same scale. Here, the CAT scale assesses positive affect using responses to questions such as whether the responder liked their classes or roommates or whether they liked their social life. Negative affect, however, is assessed using responses to questions such as feeling angry, feeling worried about academic performance or intimate relationships, or feeling lonely.

**Table 2:** Mental well-being attributes and social media platform usage of participants who do and do not use these tools for their mental health needs (N=101).

Social media for mental health disclosure and support^a^				Used		Did not use
**Academic background, n (%)**						
	Undergraduate^b^			82 (82)		82 (72)
	Graduate^b^			17 (17)		27 (27)
	Engineering or computing major			59 (59)		42 (42)
**Mental well-being scores, mean (SD)**						
	Positive affect^c^ (CAT^d^)			16.6 (3.9)		23.1 (2.6)
	Negative affect^c^ (CAT)			31.8 (7.4)		24.9 (3.5)
	Homesickness^c^ (CAT)			24.5 (5.3)		18.7 (3.8)
	Overall adjustment^b^ (CAT)			79.4 (10.7)		83.5 (9.1)
	Self-esteem^c^			18.6 (5.5)		29.8 (3.4)
	Perceived Stress Scale^c^			25.5 (5.4)		16.8 (6.5)
	General Anxiety Disorder-7^e^			9.3 (4.6)		5.1 (4.3)
	Emotional or informational support^b^			2.2 (0.5)		3.4 (0.9)
	Tangible support^b^			3.1(0.7)		3.7 (0.4)
	Affectionate support^b^			2.9 (0.8)		3.5 (0.7)
	Positive social interactions			3.6 (0.5)		4.1 (0.3)
	Medical outcomes study ^b^			2.9 (0.6)		3.6 (0.7)
**Platform use, n (%)**						
	Facebook			66 (66)		69 (69)
	Twitter			40 (40)		42 (42)
	Snapchat^b^			44 (44)		33 (33)
	Instagram^b^			41 (41)		32 (32)
	YikYak^d^			41 (41)		24 (24)
	Reddit^c^			36 (36)		21 (21)
	Tumblr^d^			37 (37)		18 (18)
**Characteristics of social media use, n (%)**						
	**Time spent on social media (hours/day)**					
		4-6^d^	55 (55)		37 (37)	
		1-3^c^	26 (26)		43 (43)	
**Purpose of social media use, n (%)**						
	Communicating with friends, family^b^			68 (68)		56 (56)
	Staying updated on friends’ activities^d^			41 (41)		76 (75)
	Finding people (old friends, classmates)^c^			33 (33)		58 (57)
	Instant access to information on social media^c^			21 (21)		48 (48)
**Connection strategy (FBI^f^), mean (SD)**						
	Initiation^b^			1.9 (0.9)		3.2 (0.6)
	Social information–seeking^c^			3.8 (0.5)		2.2 (0.7)
	Maintaining^b^			2.3 (0.8)		4.1 (0.7)

^a^Differences are statistically significant based on Mann-Whitney *U* tests followed by false discovery rate correction for multiple pairwise comparisons.

^b^*P*<.05.

^c^*P*<.01.

^d^CAT: College Adjustment Test.

^e^*P*<.001.

^f^FBI: Facebook Intensity Scale.

#### Social Media Use

Next, among the participants who used and did not use social media for mental health disclosure and support, there were differences in social media use levels and the various purposes behind its use. As shown in Table 2 and based on Mann-Whitney *U* tests and false discovery rate correction for multiple pairwise comparisons, Facebook was the most popular platform for both cohorts. However, semianonymous, ephemeral, and anonymous platforms (such as YikYak, Tumblr, and Reddit) were more actively used by those using social media for mental health help. Tumblr, for instance, was found to be used 105% more frequently in this group than in the other groups (*P*<.001), whereas Reddit was used 71% more frequently (*P*<.01).

Participants who derived value in using social media for mental health help also reported using social media more frequently (56/101, 55.4% reported using them 4-6 h a day) and for communicating with friends and family (21% more; *P*<*.*05). However, the other cohort used these platforms more often to stay updated about friends and find people (42-45% more; *P*<*.*01) and for accessing instant information (56% more; *P*<*.*01). Perhaps because of more frequent participation in social media and use of the platforms for social exchange, students in the former cohort felt encouraged to seek mental health help in an environment they already frequent. The individuals in this cohort also seem to identify with social media use for its social affordances, in contrast to the other cohort who used them for more informational purposes.

Finally, the 2 cohorts used distinct connection strategies or relational communication activities on Facebook, as included in the Facebook Intensity Scale. Social media mental health help seekers used Facebook more for information seeking (73% more; *P*<.01) than those who did not. However, they initiated fewer new connections (41% less; *P*<.05) and engaged less in the maintenance of social capital (44% less; *P*<.05). This aligns with the findings above regarding their desire to utilize these platforms to obtain information from, and communicate with their existing network, compared with nonhelp seekers who may have more proclivity to seek new friendships.

### Observations From the Interviews

Follow-up interviews were conducted following the survey. Our interview sample was heavily biased toward engineering or computing female freshmen students (8/11, 73% female; 8/11, 73% engineering or computing major; 8/11, 73% freshmen). In the remainder of this subsection, we describe the major themes that emerged from the qualitative analysis of the interviews with the students.

#### Why and How Social Media is Used for Mental Health Help

##### Engaging in Candid Self-Disclosure

Interviewed students found social media platforms to be places they visited to seek a break from stressful experiences. They also noted the value of social media as a platform to vent and commit to mental health concerns. Students reported *talking out* their frustrations with friends, posting rants on Facebook, or leaving venting voicemails on others’ WhatsApp accounts. For some, just the release of their frustrations to a friend or family member made them feel better:

I can vent and share concerns with people, like classmates, where we can both get their frustrations out.

Freshmen, female, mechanical engineering major

Friends on social media also help by reminding me of bigger picture things; I just feel like it makes me feel better.

Freshmen, female, mechanical engineering major or computer science minor

Some other students identified the value of self-disclosing to someone who dealt with the same type of stressor. Occasionally, students liked pep talks and thought they encouraged them and helped built their self-esteem:

I love the random moments of connection with people. Good to see what and how everyone else is doing.

Sophomore, male, psychology major

##### Mitigating the Feelings of Isolation

Interviewed students also identified companionship as a way social media satisfied their mental health needs. For most, this is just the feeling of not being alone. Having someone *out there*, or having someone there to listen to them, often helped the students relieve some of the stress and anxiety they experienced from college life. For instance, on social media, this type of companionship support can come from people simply *liking* or commenting on a post they wrote:

Often I find just the ability to connect to another person to be grounding, um if like I know them. You know, kind of assuage feelings of isolation that can spiral out and escalate the level of stress because you like you are in your own bubble and if you inside and this amplifies your feelings internally [...] you do not have external stimulus to bring you back and level you out. Just having a person on social media listen to you is really helpful.

Senior, male, economics major

##### Receiving Informal Help

Interviewed students also found social media helpful for mitigating mental health challenges because of the casual nature of support, advice, and help they provide. They viewed phone calls and texts as more pressing than a Facebook or WhatsApp message, and they preferred the more informal *respond whenever you get the chance* approach offered by most social media sites. They noted that this informality helps reduce stress and improve their mood in that they do not feel they are pestering or pressuring their friends or family to respond. In addition, they felt that they themselves were not obligated to respond to a social media message from a peer unless they wanted to:

Whenever I wasn’t feeling well, I could go onto Facebook and talk to my friends. I found [Facebook] really beneficial because it would kind of be sporadic and not really something I could articulate in a phone call because it wasn’t a very pressing matter or wasn’t as intense as the feelings of being upset. But then I could talk to people over Facebook and it might take a couple hours for them to respond, but when I did get the messages they were really helpful [...] I could reach out to a couple people and talk to three close friends from back home just to see how they were doing, get that off my mind, and then some responded and some didn’t. I think people feel more obligated to respond to a text immediately, but on Facebook they can say, ‘Sorry I had three tests. But are you ok now?’ and it was completely fine.

Freshmen, female, biomedical engineering major

Students also recognized the informational and tangible support and help social media platforms provide, specifically, advice, guidance, and suggestions as well as assistance with any problems they may face. They reported that although talking to their family and friends may help get advice on a relationship problem, they may ask peers on social media to help with homework assignments. Through such informational and tangible support, they recognized the casual help necessary to solve whatever problem they are facing:

I asked some friends on Facebook to help me with a project that I needed, like, people to act for and, like, they came through and they were so amazing, and I was like ‘Yes, thank you. You guys were here for me to pull me out of this anxiety.

Sophomore, female, psychology major

#### Platform Affordances and Mental Health Help

Next, our in-person interviews sought information on what affordances and features of existing social media platforms were found to be invaluable or detrimental to meeting mental health needs. Interviewed students also included their thoughts and opinions on the affordances they felt could make these sensitive disclosures and support seeking better.

##### Anonymity

The social media feature that consistently emerged in our interviews was anonymity. Students expressed both enthusiasm and concern regarding the utility of this feature. Support for the feature ranged from its ability to allow disclosure around stigmatized topics to promote quality and honest exchange: they felt that anonymous accounts eliminate components that could make a person easily identifiable (eg, name, email, and photos) or those that could trigger feelings of inadequacy in users:

I think anonymity is really important to a lot of people because like you said of the stigma behind it [...] If you have the option to be anonymous, you could even just pick a username. So if you were talking to one person, you could continue to talk to them, be able to identify them, but not know who they are on campus.

Freshmen, female, biomedical engineering major

At the same time, some of the other interviewed students felt anonymity would lower accountability on an issue that is sensitive in nature and can lead to counterproductive outcomes for mental wellness. They also brought up issues with not being able to *connect* with other users on anonymous social media websites, saying that they did not know enough about the anonymous users to feel any kind of emotional connection. They also consistently felt that a unique identifier for users would be a desired feature that could balance the pros of anonymity and the pros of having an identified account. Such user profiles, including information about academic year, area of study, or hobbies, can not only provide some context for each user but also create a means for other users to feel connected to them and want to engage with them within the tool:

I feel anonymity tends to lead to problems of lack of accountability and some people will use that to be funny in sort of a mean way.

Freshmen, women, business major

##### Trust

Trust was recognized by the students as an important construct for social media platforms, enabling mental health disclosure and support. They advocated for mechanisms that can enhance trust, such as the ability to learn more about the help seekers and providers and to curb the dissemination of illegitimate or inadvertent disclosure of personal information to web-based audiences:

I think it is nice for people to be able to voice their problems on an anonymous social support site, you know, to feel like they won’t be judged. But can you trust them? Nothing stopping people from using other people’s names in posts.

Freshmen, female, biomedical engineering major

##### Interpersonal and Collective Interactions

Interviewed students discussed a variety of different provisions for social interaction that could be beneficial for social media–based mental health disclosure and support. Generally, they felt that conversations could be more genuine and candid in a discussion board format instead of private direct messages. Private messages could increase the risk of getting bullied around disclosures as sensitive to mental health. In a discussion board setting, these risks are reduced because of the collective attention of several individuals. Students also recognized the value of smaller-sized support groups, where individuals might be more involved and committed to helping others:

I think one of the things that helps is smaller groups. I think if there is an amount of group separation, while you get access to less people as a support network, I think you get people who are more tailored to be a support network. This is mostly from my experience with Reddit [...]. People will talk about their problems and there are certain communities on Reddit, especially a lot of times, smaller communities that are super helpful and super receptive to that sort of thing. But there are other communities where, because of the size that they are because of the nature of the community, they are super unreceptive and super hostile.

Freshmen, women, business major

##### Mental Health Interventions

Finally, the interviewed students talked about explicit interventions and provisions that could better support mental health disclosures on social media platforms. These interventions could include dedicated content catering to different needs around mental health concerns (personal, academic, etc) as well as specific communities where individuals could go to seek help and advice:

A little “help me” button will be great, like, if you’re feeling particularly stressed or want help (like can’t quite get this problem). I’d also like different levels of help “what should I do?.” [...] There could be a specific area [for mental health], so if you don’t want to be looking at it, it won’t bog you down.

Freshmen, female, mechanical engineering major or computer science minor

Students also recognized the need for mechanisms to ensure rigorous security and privacy of the participants and balance the urgency for help seeking and receiving. Some students also brought up the challenges of building communities of people living with mental illness, indicating that they can amplify negative feelings that are detrimental to well-being:

Probably something also to control if you have a lot of people that are under a bunch of stress. If you have a bunch of people with the same kinds of problems in a closed space, there’s going to be an issue eventually. Like, it could be two people really, really upset, and it could bring them both down instead of up.

## Discussion

### Principal Findings

Our survey revealed that mental challenges such as stress and anxiety are fairly pervasive in the university students we studied—70.3% (71/101) of the participants indicated that they felt stressed or anxious recently. Half of them (52/101, 51.5%) also reported having appropriated some social media platforms for self-disclosure or help seeking, indicating the pervasiveness of this practice. Taken together, the survey results indicate that individuals who tend to use social media for mental health disclosure or social support were already challenged by heightened mental health concerns. They also seemed to be less adjusted to college life, with lower access to social support in offline settings. This might explain their tendency to utilize web-based tools for this purpose: previous literature has indicated that social media can provide a great deal of social and emotional support [33,37,38]. A lowered sense of self-esteem may also explain why these participants appropriated social media for their mental health needs. Previous work has revealed that using platforms like Facebook can boost self-esteem and self-worth [31]. Through our interviews, we obtained deeper insights into the initial observations. We identified specific academic, personal, and social life stressors, motivations behind social media use for mental health needs, and specific platform affordances that helped or hindered this use. Students argued against complete anonymity in platforms for mental health help, recognized the benefits of connecting informally with peers with similar challenges, and advocated the need for privacy and boundary regulation mechanisms in social media platforms supporting this use.

### Study Implications

In light of the ongoing crisis of mental health in college campuses [39], this study, combining the insights from the survey as well as the follow-up interviews, provides important insights regarding the role of social media in supporting mental health needs of students.

#### Mental Health Help via Social Media

Our study reveals that social platforms gave students the ability to find support while still maintaining some level of informality, anonymity, and privacy. Essentially, this benefit of social media disclosures of mental health concerns aligns with what has been noted in the offline context by Jourard [40]: “self-disclosure is a basic element in the attainment of mental health” and sharing narratives, stories, and experiences in written form can promote candid self-disclosure of difficult, stigmatized conditions [41]. In many ways, this student population’s mental health help-seeking behaviors, as reflected in the survey as well as the interviews, align with the observations derived from studies of the general population, such as depressed individuals seeking others out on Reddit [42,43], Instagram [37], or Twitter [44,45]; sexual abuse survivors self-disclosing about their experiences on Reddit [46]; individuals recovering from substance use appropriating online health forums [47,48]; or eating disorder patients engaging in recovery-related self-disclosure on Tumblr [49].

#### Informing Counseling Efforts and Campus Administration

Our interviews also provided insights into the practices and motivations behind students’ use of social media for mental health. It also helped us identify specific affordances provided by these platforms that were particularly facilitative of this practice. Interviewed students further identified many benefits of social media platforms for mental health disclosure and support seeking. This included provisions for both casual and emotional help, informal and private mechanisms to vent difficulties in sharing feelings, helping seek feedback on dealing with specific academic and personal life-related stressors, and the ability to connect with a large on-campus commiserating student community. Together, these pieces of information can be highly beneficial to campus counseling centers in understanding attributes of the mental health of students and the pervasiveness of these challenges, especially given the limited counseling workforce and resources available in many college campuses relative to student needs. Observing what student users are discussing in a web-based social support platform could offer these professionals insights into the types of problems students are currently facing. In addition, observing how students use the platforms and how they interact with others could provide a better understanding of how students manage their problems and handle social interactions. This information could potentially help counseling centers with their services and strategies to better reach students and better fit their needs.

### Recommendations for Social Media–Based Support Technology Design

Our findings also have implications for the design of next-generation and improved social media support tools that can address mental well-being issues in college students. These interventions can also take advantage of the affordances of both current social media and their social support mechanisms to better help students tackle mental health challenges.

#### Anonymity and Identifiability

Our survey reported high usage of anonymous and ephemeral social media sites among students seeking mental health help on the internet; it was 70% more than that among the students who did not use social media for mental health needs. However, somewhat surprisingly, the interviewed students did not recognize the unanimous utility of having anonymity as a helpful feature. Although they also mentioned the potential risks of using a fully identified platform for the purpose, semianonymity seemed to be an agreed-upon compromise. This was because semianonymity allowed moderate accountability and the ability for students to connect with like-minded peers on campus, thus helping establish credibility and trustworthiness, but could prevent disclosing information that may be personally identifiable. We note that this type of trade-off or dichotomy between anonymity and identifiability is well-documented in the literature, although not in the context of mental health help seeking. As Cutrona [50] notes, “how to disclose enough of one’s misery to gain the benefits such revelations can provide, without disclosing in such a way or to such an extent that it will drive others away.” This dichotomy also reflects interviewed students’ concern that anonymity can encourage offensive behaviors, bullying, or harassment toward those who might be in crisis and in need of help around a stigmatized condition. College student–oriented or campus-restricted Reddit-like forums may thus be designed to better cater to student-specific needs around mental health issues identified in our study, such as schoolwork, relationships, college life, or career paths.

#### Trustworthiness and Credibility

On a related note, interviewed students also recognized trust as an important element driving their desire to obtain mental health help on social media. As Altman described in his work on interpersonal exchange [51], sensitive self-disclosures modify a *self-boundary* (the boundary around the individual) and a *dyadic boundary* that ensures the discloser’s safety from leakage of information to uninvited third parties. Both boundaries are influenced by interpersonal factors such as the level of trust in the disclosure target. In other words, trust in sensitive disclosure is of utmost importance. This is because mental health help seeking can impose certain risks to disclosers. This may include becoming defenseless and unguarded, receiving negative feedback, or what Wenburg and Wilmot termed the *reverse halo effect* [52] (the possibility of revealing a weakness can lead the disclosed-to person to generalize about other weaknesses of the discloser). Therefore, it is understandable that surveyed students, who used social media for mental health help, used connection strategies that were more informational and were less likely to initiate new ties.

Thus, to make trust a built-in feature, social and reputation markers may be included in campus-specific web-based communities of support. For example, user responses can be appropriately weighed in terms of trustworthiness. Such markers could also include point systems for the number of helpful comments or advice provided, badges of community service toward mental health help, or dynamic up- and down-voting mechanisms to evaluate the quality of responses in real time. Interpersonal trust information can also be incorporated by assessing the strength of historical social interactions between 2 individuals.

#### Peer Support

Our survey identified that students who used social media for mental health help had lower offline social support than those who did not (11% less). It is not surprising that many students, in the interviews, reported social connectedness facilitated by small web-based groups to be one of the most prominent motivations behind their desire to use social media platforms for mental health. Why students seek social connectedness can spring from specific needs related to mental health challenges? The social comparison theory [53] states that one turns to those that are similar to themselves in terms of the experience because they are presumed to provide the most relevant information for making an accurate judgment of how to respond. Furthermore, participation in smaller groups can enhance the perceived benefits of social connectedness that can allow people to vent or open up more comfortably. As a design feature, social media platforms could, therefore, incorporate the ability to start on-demand interpersonal or smaller group chats, aside from allowing content sharing in a larger community. These informal but more focused disclosure mechanisms could promote a secure way of social bonding, mitigating feelings of isolation and engaging in mutual commiseration.

#### Boundary Regulation

Students’ desire for small group disclosures of mental health concerns can also stem from the challenges of context collapse and difficulties in boundary regulation and managing privacy [54]. Therefore, many web-based platforms allow people to better regulate the boundaries of their self-disclosures, primarily via privacy access control mechanisms. However, as Ellison et al [55] noted, “privacy behaviors on SNSs are not limited to privacy settings.” Naturally, individuals adopt a variety of other techniques for information regulation, such as the creation of multiple identities [56] or adjusting profile visibility [57]. Our interviewed student cohort identified value in small group disclosures on the web as a way to circumvent the issues of context collapse and regulate information disclosure boundaries. Along these lines, social media platforms could create private and topic-oriented spaces to discuss stigmatized topics, wherein individuals could seek and provide mental health help from time to time without disrupting their activities elsewhere on the platform or compromising their privacy in other discourses.

#### Web-Based Interventions

Finally, interviewees shared many elaborate thoughts about mental health support mechanisms and interventions that could be built on future social media platforms. These ranged from *help me* buttons to elaborate information on how to cope with stress and mental health crises and structured provisions not only for help seekers but also providers. Accordingly, we described 2 such support-based interventional strategies: (1) platforms could issue public service announcements, emotionally uplifting content, and informational pointers to support seeking individuals, such as an appropriate hotline on or off campus. This would increase students’ likelihood of being exposed to coping strategies or supporting resources, and (2) social media platforms can pair up students experiencing particular college and academic life stressors with others who have been successful in addressing these challenges. This would encourage informal ways of seeking and providing peer support.

### Limitations

Our work has some limitations that we acknowledge. The first relates to the generalizability of the findings. Although our survey can be applied to any college campus, the specific findings apply only to students at the university we study. From the survey data, we found a large fraction of students who self-report and score to be challenged by mental health concerns, such as stress and anxiety. We note that these findings may not apply to the broader student community in the United States or elsewhere in the world. In particular, we also note the limitations of some of the survey scales used, such as the MOS social support survey. This scale captures offline social support, and we used this scale in this study to understand the extent of availability of offline social support to students and how students with less or more offline social support appropriate social media to supplement and complement it. Future work can develop and adapt a scale that would serve as a web-based counterpart to the MOS survey to corroborate the extent to which web-based help-seeking behaviors of college students mirror their offline needs and support.

In addition, there are possible limitations of social media as a solution to help cope with mental health challenges—a dimension that was not explored through our survey or interviews. Although social media websites offer students a low-pressure, informal way to seek support, as our study revealed, presumably, they do not provide the same type of direct social support as an in-person interaction would. Therefore, we suggest caution in the interpretation of this study’s findings. That is, we recognize that social media tools cannot be used as standalone counseling or treatment mechanisms, which is particularly true for the interventions outlined above. Essentially, given the potential of social media to support students’ mental health needs, as revealed in this paper, it could act as a catalyst for in-person support, providing a way for students to meet other students on the web in an informal, real-time setting without feeling stigmatized.

### Conclusions

As suicide is the second leading cause of death on college campuses, addressing the mental health of college students is extremely important. Motivated by this observation, in this paper, we present a comprehensive study to investigate the role of social media in meeting students’ mental health needs. Our survey of students at a large public university in the southeast of the United States revealed extensive use of social media platforms for seeking mental health help; 51.5% (52/101) of the surveyed students reported this use. We followed up with in-person semistructured interviews with students to identify the intent and motivation behind these practices and the benefits and challenges they perceived to exist on social media platforms toward mental health help seeking. We found that students turned to social media to seek help because they could vent and engage in candid self-disclosure and mitigate feelings of isolation, all in an informal, semianonymous setting. We believe our findings provide fresh insights into how social media–based interventions and provisions for support can be built to improve college students’ mental well-being as well as to help inform campus mental health counseling and mitigation efforts.

## Appendix

Multimedia Appendix 1 Recruitment flyer used for online advertisements of the study.

Multimedia Appendix 2 Interview guide outlining semistructured questions for participants.

Multimedia Appendix 3 Codebook used for qualitative coding of the interview transcripts.

## Abbreviations

CAT: College Adjustment Test
MOS: medical outcomes study
