# Supplementary material for: Understanding the Role of Social Media–Based Mental Health Support Among College Students: Survey and Semistructured Interviews
Source: JMIR Ment Health. 2021 Jul 12;8(7):e24512. doi: 10.2196/24512 (PMC8314152; doi:10.2196/24512)
Supplement: Multimedia Appendix 1 [file mental_v8i7e24512_app1.pdf]

Appendix A

Recruitment

We will recruit current Georgia Tech students, both undergraduate and graduate levels, ages 18-24. Our online survey will be administered across multiple online channels in order to reach a large number of Georgia Tech students. The survey will be posted on Facebook, and requests will be sent to the appropriate persons to post the survey on Reddit (specifically, the Georgia Tech subreddit) and the Georgia Tech Counseling Center website. Participants recruited via Facebook, Reddit, or the GT Counseling Center will be compensated with a $10 Amazon gift card.

Below is a draft of the email we will use to request access to post our survey via Reddit and the GT Counseling Center website:

Subject: Study on student well-being and social media behavior

I am a Georgia Tech master's student, working with Prof. Munmun De Choudhury, conducting a study to better understand the psychological well-being and social media usage/behaviors of current Georgia Tech students. The ultimate goal of this research is to identify how an online platform could be used to offer social support to Tech students who are facing mental health concerns. The study consists of an online survey, and we would love the opportunity to reach more Georgia Tech students by having it posted on [Reddit, Counseling Center website]. If this is something you think could be possible, please reach back out to me. I'd be happy to answer any questions or concerns you might have.

Thank you, Piper Vornholt

Below is a draft of the survey posting for recruitment via Facebook, Reddit, and the GT Counseling Center website:

Title: Help researchers understand your well-being, college experience, and social media usage. Participants will receive a $10 Amazon gift card.

We researchers at Georgia Tech are conducting a study to identify the psychological well-being, college experiences, and social media behaviors of current Georgia Tech students, ages 18-24. The study involves taking an online survey that consists of multiple measures of behavioral and social media usage. The survey is estimated to take no more than 45 minutes to complete, and participants will receive a $10 Amazon gift card as compensation. If you are interested, please click here to begin the survey: [insert survey link]. Feel free to share this survey with anyone else who may be interested. Thank you!
