# Supplementary material for: Understanding the Role of Social Media–Based Mental Health Support Among College Students: Survey and Semistructured Interviews
Source: JMIR Ment Health. 2021 Jul 12;8(7):e24512. doi: 10.2196/24512 (PMC8314152; doi:10.2196/24512)
Supplement: Multimedia Appendix 2 [file mental_v8i7e24512_app2.pdf]

Appendix B

Discussion Guide for Semi-structured Interview

(everything below is to be read by the researcher to the participant)

Thank you for joining me today. I am a student researcher, and I am talking to Georgia Tech students to get a better understanding of how they use social media to gain social support for coping with stress and anxiety. I am going to ask you several questions about your social media usage, how you find social support, and how you manage any stress or anxiety you may have, and I will be taking notes and recording audio as we speak. Please remember that you may skip any questions that you do not feel comfortable answering, and you may take a break from this study at any time. Do you have any questions before we get started?

To start off, please tell me a little about yourself…

- What year are you? (Freshmen, Sophomore, etc.)
- What is you academic discipline/area of study?

What social media sites and apps do you like to use?

- What is the primary source that you use for social media? (laptop, phone, tablet, etc.)
- What attracts you to social media?
- Do you like to use anonymous or non-anonymous social platforms? Why?

Do you experience any stress or anxiety from college life? Tell me about that.

- What methods do you use to manage your stress and/or anxiety?

What role does social media play in helping you cope with your stress and/or anxiety?

- Identify the role of different social platforms (anonymous and non-anonymous) that you may be using.
- What aspects/features of the social media sites you use help you relieve stress?
- What aspects/features help you relieve anxiety?

Have you used social media to find support for coping with stress or anxiety?

- Who do you find support from OR who you do you prefer to find support from (friends, peers, anonymous users)?
    - Why?

   - What are the benefits of finding support from friends/peers/anonymous users?
- How do you find this support? Describe in what ways social media has helped you obtain social support.
- Describe in what ways social support has helped you cope with stress and/or anxiety.

Those are all of my questions. If you have any follow up questions or any additional thoughts, please do not hesitate to contact me. Thank you so much for your time, and I hope you enjoy the rest of your day. *(give participant compensation gift card)*
