# Supplementary figures and images for: Understanding the Role of Social Media–Based Mental Health Support Among College Students: Survey and Semistructured Interviews
Source: JMIR Ment Health. 2021 Jul 12;8(7):e24512. doi: 10.2196/24512 (PMC8314152; doi:10.2196/24512)

## Appendix C

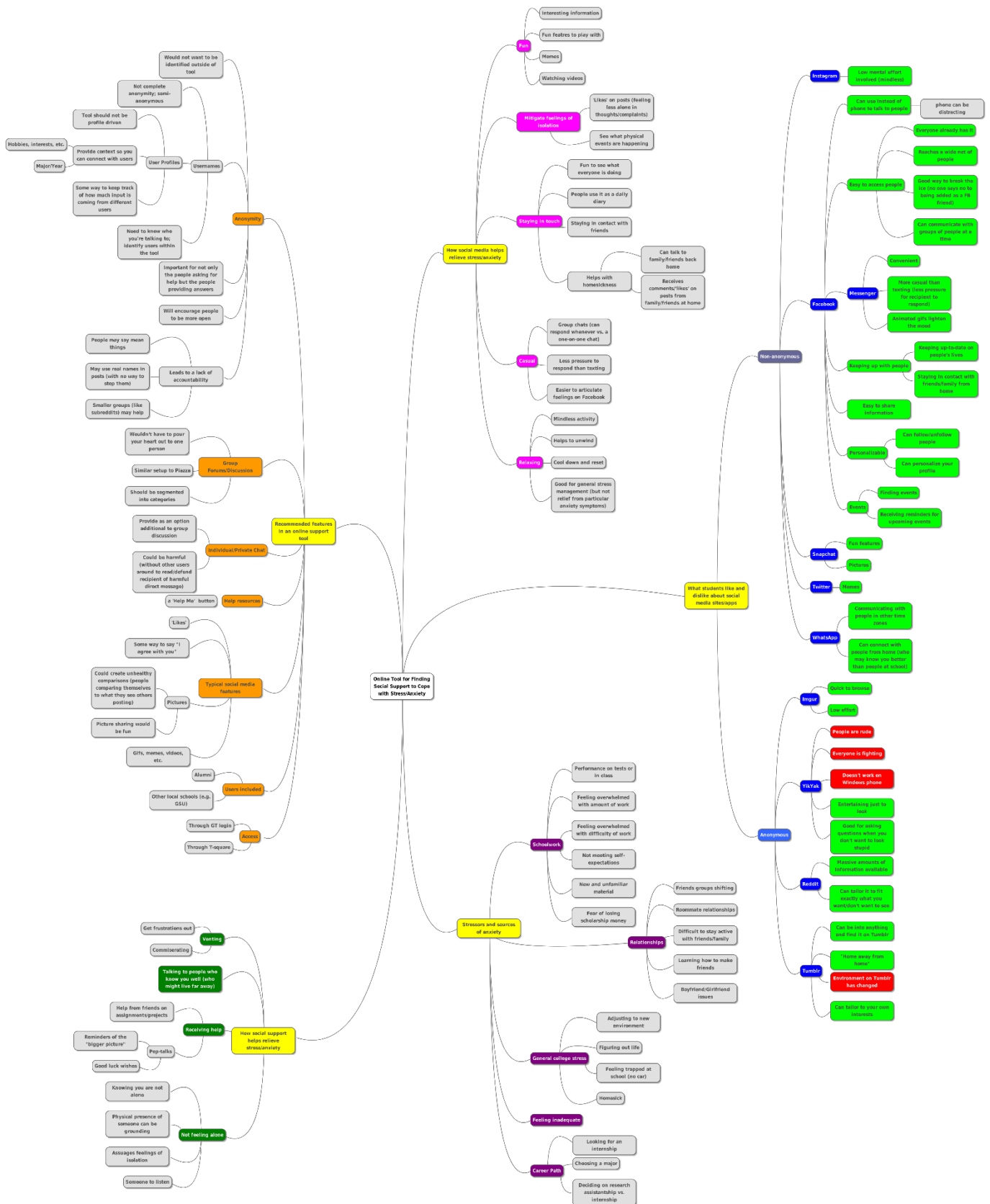

Supplement: Multimedia Appendix 3 [file mental_v8i7e24512_app3.pdf]
